# Serum Level of Soluble Receptor for Advanced Glycation End Products Is Associated with A Disintegrin And Metalloproteinase 10 in Type 1 Diabetes

**DOI:** 10.1371/journal.pone.0137330

**Published:** 2015-09-01

**Authors:** Alan C. H. Lee, Joanne K. Y. Lam, Sammy W. M. Shiu, Ying Wong, D. John Betteridge, Kathryn C. B. Tan

**Affiliations:** 1 Department of Medicine, University of Hong Kong, Hong Kong, China; 2 Department of Medicine, Royal Free & University College London Medical School, London, United Kingdom; National Center for Scientific Research Demokritos, GREECE

## Abstract

**Background:**

The receptor for advanced glycation end products (RAGE) is involved in the pathogenesis of diabetic complications, and soluble forms of the receptor (sRAGE) can counteract the detrimental action of the full-length receptor by acting as decoy. Soluble RAGE is produced by alternative splicing [endogenous secretory RAGE (esRAGE)] and/or by proteolytic cleavage of the membrane-bound receptor. We have investigated the role of A Disintegrin And Metalloproteinase 10 (ADAM10) in the ectodomain shedding of RAGE.

**Methods:**

Constitutive and insulin-induced shedding of RAGE in THP-1 macrophages by ADAM10 was evaluated using an ADAM10-specific metalloproteinase inhibitor. Serum ADAM10 level was measured in type 1 diabetes and control subjects, and the association with serum soluble RAGE was determined. Serum total sRAGE and esRAGE were assayed by ELISA and the difference between total sRAGE and esRAGE gave an estimated measure of soluble RAGE formed by cleavage (cRAGE).

**Results:**

RAGE shedding (constitutive and insulin-induced) was significantly reduced after inhibition of ADAM10 in macrophages, and insulin stimulated ADAM10 expression and activity. Diabetic subjects have higher serum total sRAGE and esRAGE (p<0.01) than controls, and serum ADAM10 was also increased (p<0.01). Serum ADAM10 correlated with serum cRAGE in type 1 diabetes (r = 0.40, p<0.01) and in controls (r = 0.31. p<0.01) but no correlations were seen with esRAGE. The association remained significant after adjusting for age, gender, BMI, smoking status and HbA1c.

**Conclusion:**

Our data suggested that ADAM10 contributed to the shedding of RAGE. Serum ADAM10 level was increased in type 1 diabetes and was a significant determinant of circulating cRAGE.

## Introduction

Proteolytic ectodomain release is one of the key mechanisms for regulating the function of a number of cell surface proteins. The process, also known as shedding, is mediated mainly by the proteinase family A Disintegrin And Metalloproteases (ADAMs) which are type 1 transmembrane proteins [1]. Twelve members of the ADAM family display catalytic function and mediate the proteolytic cleavage of cell surface integral membrane proteins within their juxtamembrane region, releasing a soluble protein ectodomain into the extracellular space. It has been estimated that up to 4% of the proteins on the cell surface undergo ectodomain shedding and this process affects functionally diverse proteins [2]. Dysregulation of ectodomain shedding is associated with a wide range of pathological conditions, including autoimmune and cardiovascular diseases, neurodegeneration, infection, inflammation and cancer [3].

ADAM10 is one of the proteolytically active ADAM members and has been studied in particular due to its potential role in the pathogenesis of Alzheimer’s disease, since amyloid precursor protein is one of the substrates of ADAM10 [4,5]. The number of recognized ADAM10 substrates is increasing, confirming the central role of ADAM10 in many important biological processes, such as cell migration and axonal navigation, cell adhesion and regulation of immune reactions and control of apoptosis [6,7]. Recent studies using cell lines have suggested that ADAM10 is also involved in the cleavage of the receptor for advanced glycation end products (RAGE), a multi-ligand member of immunoglobulin superfamily of transmembrane cell surface molecule [8]. Activation of the RAGE axis has been implicated in the pathogenesis of diabetic vascular complications [9,10]. Soluble forms of RAGE can potentially act as decoy for RAGE ligands and have been used as treatment to block RAGE activation in diabetic animals [9]. It has been suggested that soluble forms of RAGE (sRAGE) can be produced by cleavage of cell surface receptor (cRAGE) or by alternative splicing of the RAGE gene [endogenous secretory RAGE (esRAGE)] [11,12].

Stimulation of RAGE shedding might potentially have a therapeutic value but the regulation of this process is poorly understood. The process of shedding of cell surface proteins can be constitutive and/or inducible. Shedding can be activated by G-protein coupled receptors, protein kinase C, intracellular calcium levels, membrane lipid composition and other experimental and natural stimuli [13]. Recent studies have suggested that shedding of RAGE can be induced in vitro. Proteolytic cleavage of full-length RAGE can be stimulated by Ca^2+^-ionophore ionomycin, G protein-couples receptors and by pharmacological agents like statins [14–17]. We have recently shown that insulin also increases the cleavage of full-length cell surface RAGE to form sRAGE in THP-1 macrophages [18]. Although in vitro studies have suggested a role of ADAM10 in shedding of RAGE [8], its clinical significance remains unclear and has not been investigated. In the present study, we have further determined the effect of insulin on ADAM10 expression and activity in vitro and evaluated whether there are changes in ADAM10 level in type 1 diabetes and its role in determining levels of circulating soluble RAGE isoforms.

## Methods

### In vitro studies

A human monocytic leukemia cell line, THP-1 cells (ATCC, Manassa., VA) was grown in RPMI 1640 medium containing 10% FBS at 37°C in 5% CO_2_. Cells were plated and differentiated for 72 hours before being used in experiments. The cells were then incubated with or without insulin in the presence or absence of the indicated metalloproteinase inhibitors for each experiment. To determine the role of ADAM10 in constitutive and insulin-induced shedding of RAGE, cell-surface receptors of THP-1 macrophages were biotinylated with sulpho-NHS-LC biotin (Pierce, Rockford, IL, USA). Cells were incubated with or without insulin (10 mIU/mL) for 24 hours. Cell conditioned-media was then harvested and immunoprecipitated with anti-biotin agarose (Sigma, St. Louis, MA), and analyzed by immunoblotting with anti-RAGE monoclonal antibody or streptavidin-HRP antibody (STP-HRP) (Abcam, Cambridge, UK). Experiments were repeated in the presence of GM6001 (20uM), a broad-spectrum hydroxamate metalloproteinase inhibitor; or GI254023X (20uM), an ADAM10-specific metalloproteinase inhibitor, (Calbiochem, San Diego, CA) to block the shedding of RAGE.

To further investigate the effect of insulin on ADAM10 protein expression and activity and cleavage of RAGE, dose-dependent experiments were performed. THP-1 macrophages were incubated with increasing concentrations of insulin in serum-free and PMA-free medium for 24 hours. ADAM10 protein expression was measured by Western blot and ADAM10 activity in cell lysate was determined by a fluorimetric assay (SensoLyte 520 ADAM10 activity assay kit, Fremont, CA). Cell conditioned-media was harvested for measurement of cRAGE. All experiments were repeated 3–5 times. Experiments to determine the effect of insulin on ADAM10 protein expression and shedding of RAGE were also repeated in human monocyte-derived macrophages (MDMs). Peripheral blood mononuclear cells were first isolated from healthy control subjects by density centrifugation using Ficoll (GE Healthcare Bio-Sciences, Pittsburgh, PA). Purified monocytes (2x10^6^cells/ml) were cultured in IMDM (Invitrogen, Grand Island, NY) medium supplemented with penicillin-streptomycin and 20% (vol/vol) autologous serum for 8 days to allow differentiation into monocyte-derived macrophages.

### Clinical study

Patients with type 1 diabetes were recruited from the diabetes clinic at Queen Mary Hospital. Patients with malignancy, connective tissue disease, previous history of cardiovascular disease, liver dysfunction or renal impairment were excluded. Patients on statin therapy were also excluded as statins have been shown to induce RAGE shedding [16,17]. Healthy control subjects were recruited from the community and none of them had history of chronic illness and/or was receiving regular medications. The study was approved by the Ethics Committee of the University of Hong Kong, and written informed consent was obtained from all subjects. Fasting blood samples were taken for the measurement of glucose, HbA1c, lipids, creatinine, ADAM10, sRAGE and esRAGE.

Immunoprecipitation of serum ADAM10 protein using a rabbit anti-human polyclonal antibody specific to ADAM10 (Millipore, CA) followed by Western blot analysis was first performed to prove that ADAM10 was detectable in human serum. An in-house competitive ELISA was then developed to measure serum level of ADAM10. 50ul of 50ng/ml synthetic peptide of human ADAM10 (amino acids 732–748 near the C-terminus, Millipore, CA) was first coated overnight at 4°C in 0.1M sodium bicarbonate, pH 9.6. Serum samples were one-fourth diluted with 1% BSA/PBS prior to the assay. After blocking the ELISA with 5% BSA/PBS for 3 hours, 50ul of either above 1/4 diluted serum samples or series diluted antigen standards plus 50ul of 1:50000 rabbit polyclonal anti-human ADAM10 antibody was added to wells for 3 hours incubation at room temperature. Post-secondary goat-anti-rabbit polyclonal HRP conjugated IgG (100ul, Sigma, St. Louis, MO) at 1:2000 dilution was added after 5 times of buffer washes. After further washing steps, 100ul per well of 3,3′,5,5′-tetramentylbenzidine substrate (TMB substrate kit; Pierce Rockford, IL) was added for 20mins followed with 50ul per well of 2M sulfuric acid (H_2_SO_4_) as stop solution. Absorbance reading was read within 30mins by an ELISA reader at 450nm. The standard curve was linear between 80 and 250ng/ml by using known concentrations of ADAM10 peptide as the standard to ADAM10 antibody. The average recovery was 101%, and ranged from 84 to 109% accordingly. The intra- and inter-assay coefficients of variation (CV) of the competitive ELISA were 4.1% and 7.7%, respectively.

Serum sRAGE (Quantikine; R&D systems, Minneapolis, MN, USA) and esRAGE levels (B-Bridge International Inc., CA, USA) were measured using commercially available ELISA kits according to the manufacturer’s protocol. Measurements were performed in duplicate. The intra- and interassay CV were 1.9% and 5.5% for sRAGE respectively and 3.7% and 5.4% for esRAGE respectively. There is no assay that can specifically measure soluble forms of RAGE formed by shedding. Hence, we have used the difference between total sRAGE and esRAGE as an estimated measure of soluble forms of RAGE formed by cleavage (cRAGE). Plasma total cholesterol, HDL cholesterol and triglycerides were determined enzymatically on an analyzer (Hitachi 912; Roche Diagnostics). LDL cholesterol was calculated by the Friedewald equation or measured directly if fasting triglyceride was greater than 4.5 mmol/L. HbA1c was measured in whole blood using ion-exchange high-performance liquid chromatography with the Bio-RAD Variant Haemoglobin Testing System (Bio-Rad Laboratories, Hercules, CA, USA). Estimated glomerular filtration rate (eGFR) was calculated using the Modification of Diet in Renal Disease (MDRD) Study equation.

### Statistical Analyses

Data that were not normally distributed were logarithmically transformed before analyses were made. Differences in continuous variables between diabetic patients and controls were determined by independent sample *t*-test. Pearson’s correlations were used to test the relationship between variables and multiple linear regression model was used to assess the relationships between soluble RAGE isoforms and various variables simultaneously.

## Results

**Fig 1:**
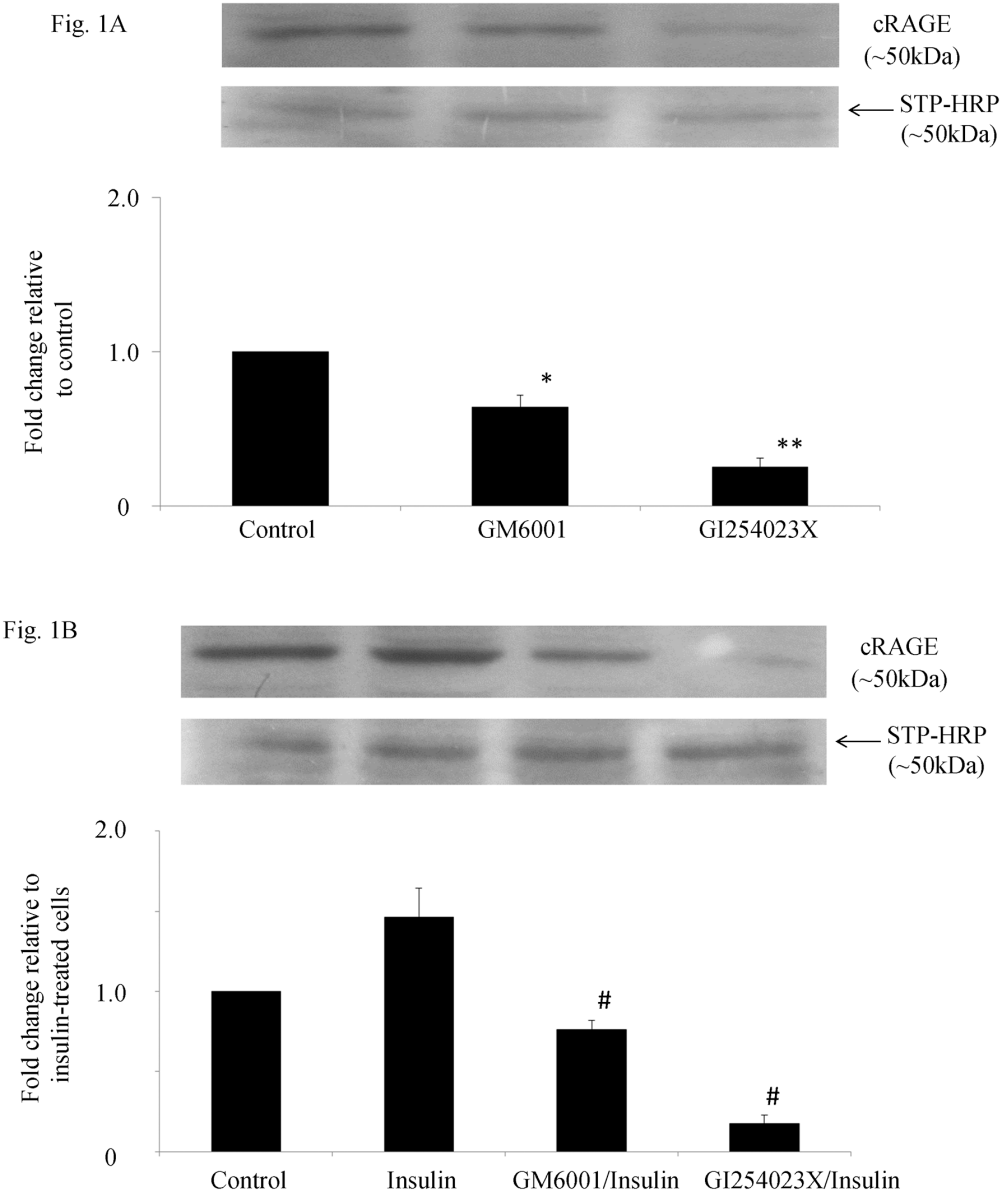
Effect of inhibition of ADAM10 on constitutive (A) and insulin-induced shedding of RAGE (B) in THP-1 macrophages. Cell surface receptors were biotinylated and cell-conditioned media was immunoprecipitated with anti-biotin agarose, electrophoresed and immunobloted with anti-RAGE antibody or streptavidin-HRP (STP-HRP) antibody. Biotinylated RAGE in cell-conditioned media (cRAGE) was significantly reduced by addition of GM6001 (*p<0.05) and GI254023X (**p<0.01) vs control cells (A). Cells were incubated with insulin (10mIU/ml) for 24 h in the presence or absence of inhibitors and biotinylated RAGE in cell-conditioned media was quantified. Data represent the mean ± SEM. ^#^p< 0.01 vs insulin-treated cells (B).

The role of ADAM10 in constitutive and insulin-stimulated shedding of RAGE in THP-1 macrophages was first investigated in vitro and the results are shown in Fig 1A and 1B respectively. Cell surface RAGE was biotinylated and the amount of biotinylated RAGE shedded into cell conditioned media represented cRAGE formed by ectodomain cleavage of RAGE. The addition of either GM6001 or G1254023X significantly reduced the amount of biotinylated RAGE in cell-conditioned media (Fig 1A). The decrease in biotinylated RAGE in cell-conditioned media was greater when the specific ADAM10 inhibitor G1254023X was used, suggesting that constitutive shedding of RAGE was blocked by inhibiting ADAM10. In cells treated with insulin for 24 hours, insulin increased shedding of biotinylated RAGE into cell-conditioned media by at least 1.5 fold and this process could also be blocked by inhibiting ADAM10 (Fig 1B).

**Fig 2:**
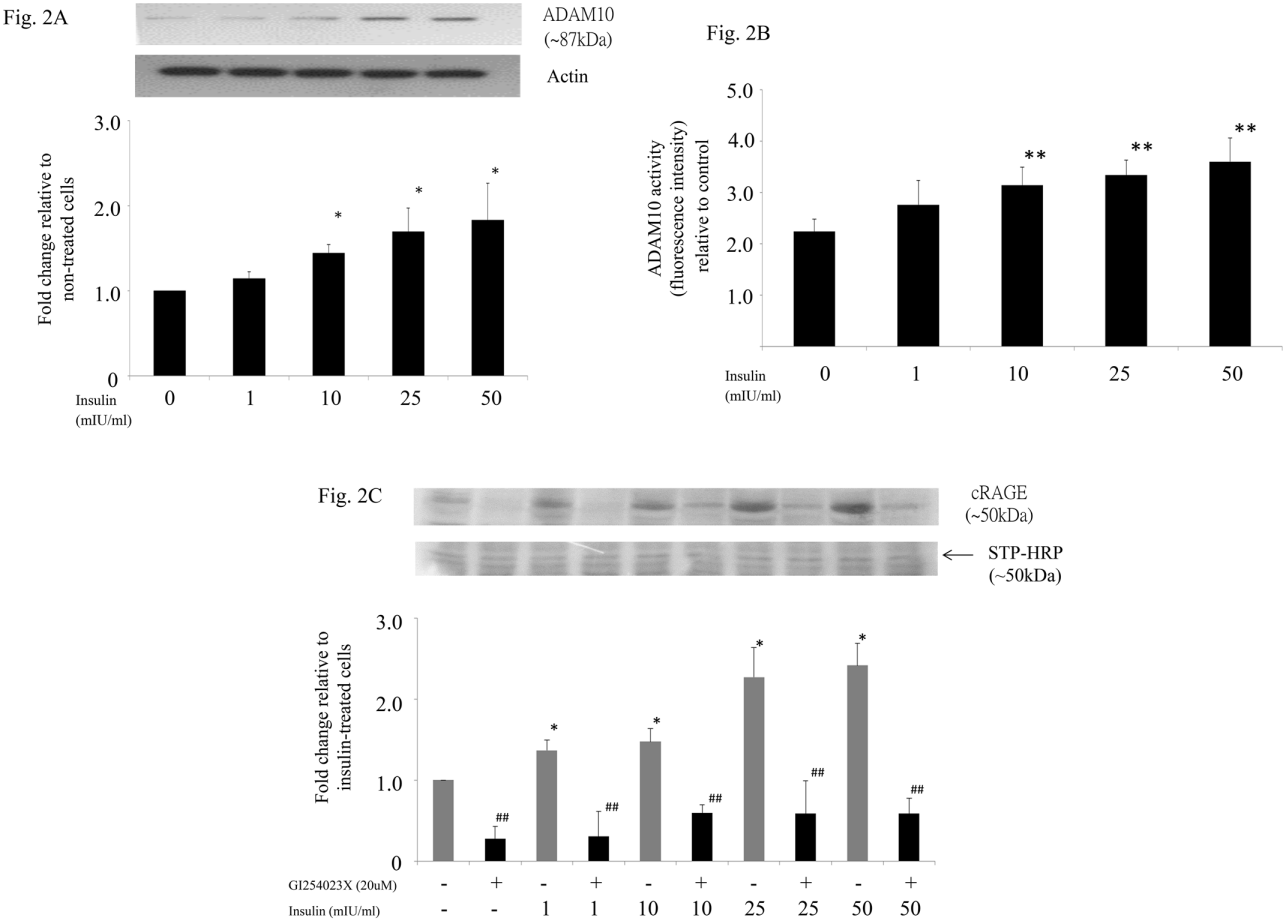
Effect of insulin on ADAM10 protein expression (A) and activity (B) and shedding of RAGE (C) in THP-1 macrophages. THP-1 macrophages were incubated with increasing concentrations of insulin (0 to 50 mIU/ml) or blank medium as control for 24 hours. ADAM10 protein in whole cell lysate was then measured by Western blot (A) and cellular ADAM10 activity was measured by fluorimetric assay (B). Data represent the mean ± SEM. *p<0.05, **p<0.01 vs control. Cell-conditioned media was harvested for quantification of cRAGE and experiments were repeated with the addition of specific ADAM10 inhibitor (C). *p<0.05 vs control, ^##^p<0.01 vs corresponding insulin-treated cells.

To further investigate whether insulin increased shedding of RAGE by stimulating ADAM10 expression and/or activity, THP-1 macrophages were incubated with increasing concentrations of insulin, and ADAM10 protein expression and activity in cell lysate was determined. Western blot analysis showed that insulin increased ADAM10 expression in a dose-dependent manner (Fig 2A). This was paralleled by an increase in ADAM10 activity in the cell lysate (Fig 2B) and shedding of cell surface RAGE into cell-conditioned media which can be blocked by inhibiting ADAM10 (Fig 2C). Taken together, our data would suggest that insulin increases shedding of RAGE by stimulating ADAM10 expression and activity. In addition, we have shown that insulin stimulates ADAM10 expression and shedding of cell surface RAGE not only in THP-1 macrophages but also in human monocyte-derived macrophages (S1 Fig).

Since our in vitro experiments suggest that ADAM10 may play a role in insulin-stimulated RAGE shedding, we have measured serum ADAM10 in a group of type 1 diabetic patients and healthy controls, and evaluated the relationship between ADAM10 and the various soluble RAGE isoforms. The clinical characteristics of the subjects are shown in Table 1. All patients were treated with multiple daily insulin injections or were on continuous subcutaneous insulin infusion. The mean total daily dose was 46±16 units. Fourteen percent of the patients had microvascular complications. As expected, patients with type 1 diabetes had higher fasting glucose and HbA1c. They also had lower plasma triglyceride and higher plasma HDL than control. Serum ADAM10 level was significantly increased in type 1 diabetic patients compared to controls (Table 1). There was no correlation between serum ADAM10 level and daily insulin dosage or age, BMI or HbA1c in the diabetic patients. Levels of serum sRAGE, esRAGE and cRAGE are also shown in Table 1. Patients with type 1 diabetes had higher levels of sRAGE, esRAGE and cRAGE than controls (p<0.01).

**Table 1:** Clinical characteristics and serum levels of ADAM 10 and soluble RAGE isoforms in controls and type 1 diabetic patients.

	Control	Type 1 DM
Number of subjects	101	102
Sex (male/ female)	42/58	40/60
Age (years)	43.2±10.2	42.1±11.1
Duration of diabetes (years)	-	17.4 ± 9.1
BMI (kg/m2)	24.2±3.4	23.1±3.5*
Waist circumference (cm)	81.3±9.9	76.3±12.4**
Smoker (%)	9.9%	9.8%
ACEI/ARB (%)	-	25.5%
SBP (mmHg)	117.2±15.2	121.8±17.2*
DBP (mmHg)	75.5±11.1	74.5±10.0
FG (mmol/L)	5.2±0.6	9.7±4.3**
HbA1c (%)	5.4±0.4	8.3±1.4**
HbA1c (mmol/mol)	35.5±4.4	67.1±15.1**
Total cholesterol (mmol/L)	4.85±0.87	4.79±1.10
Triglycerides (mmol/L)	1.00 (0.75–1.30)	0.70 (0.60–0.90)*
LDL (mmol/L)	2.76±0.77	2.67±0.91
HDL (mmol/L)	1.31±0.37	1.73±0.49**
Creatinine (umol/L)	78.7±16.5	75.3±16.3
eGFR (ml/min/m2)	90.6±17.0	93.6±16.4
ADAM10 (ng/ml)	156 (112–278)	324 (179–433)**
sRAGE (pg/ml)	802 (532–1129)	1038 (749–1217)**
esRAGE (pg/ml)	285 (210–381)	367 (269–476)**
cRAGE (pg/ml)	484 (283–796)	594 (447–812)**

Values are mean ± SD, or median (interquartile range) or percentage.

*p<0.05

** p<0.01 vs controls.

**Fig 3:**
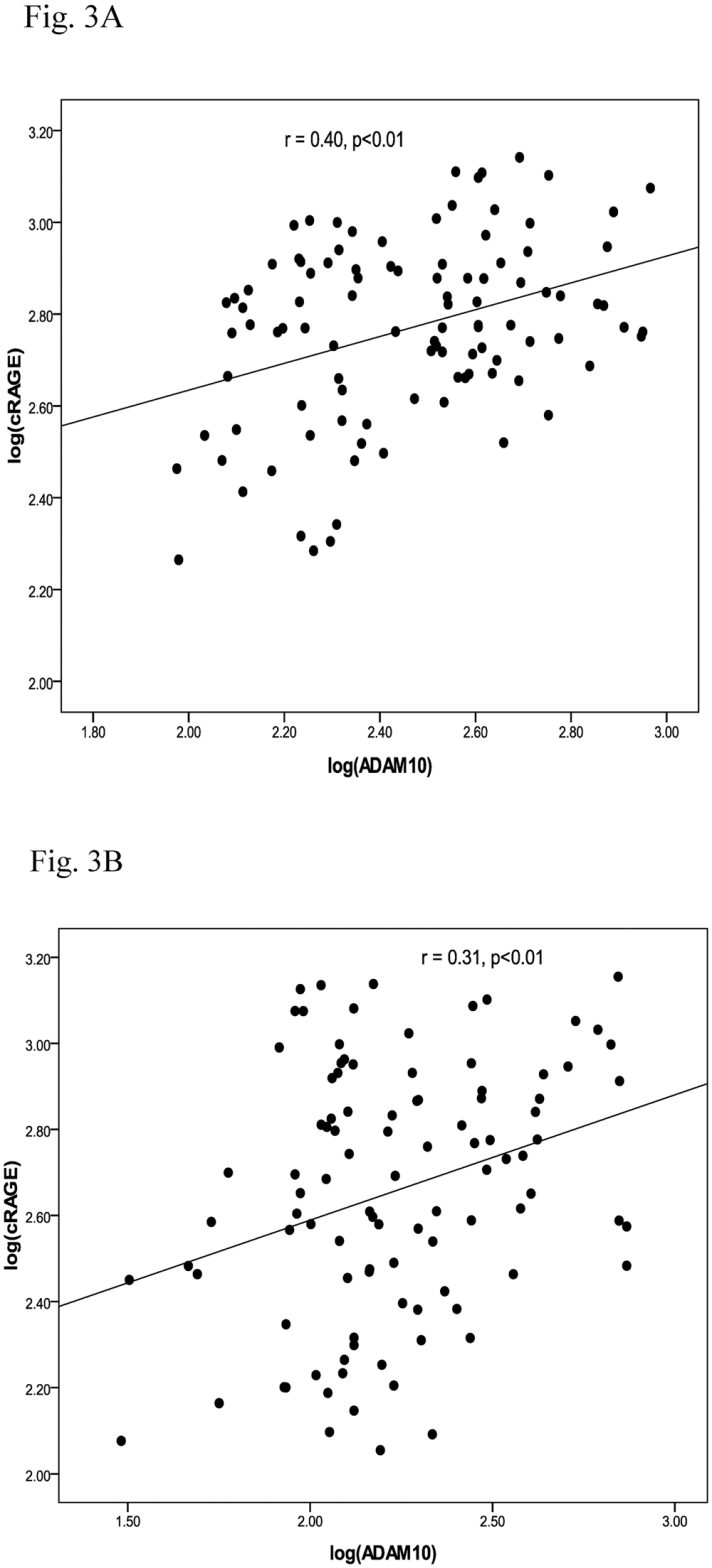
Correlation between ADAM10 and cRAGE in type 1 diabetes (A) and control (B).

Correlation analysis was performed to determine whether there was any relationship between ADAM10 and the various isoforms of soluble RAGE. Serum ADAM10 correlated with sRAGE in both diabetic patients (r = 0.30, p<0.01) and controls (r = 0.30, p<0.01). Hence, ADAM10 was associated with the total amount of soluble forms of RAGE in the circulation. Further analysis showed that ADAM10 only significantly correlated with cRAGE (Fig 3A and 3B) but not with esRAGE. Multiple linear regression analysis was performed to determine whether ADAM10 remained a significant independent determinant of serum cRAGE in patients with type 1 diabetes after adjusting for age, gender, body mass index, smoking status and HbA1c. Serum ADAM10 was an independent determinant of serum cRAGE in patients with type 1 diabetes (p<0.01), and accounted for 21% of the variation in serum cRAGE. Further adjusting for duration of diabetes and insulin dose did not change the results.

## Discussion

The ADAMs are a large, widely expressed, and developmentally regulated family of proteins and ADAM-mediated shedding of integral membrane proteins is essential for a number of biological processes important for embryonic development and tissue homeostasis[1,2]. ADAM10 and ADAM17 are the main sheddases responsible for ectodomain shedding and changes in the expression levels of ADAM and their dysregulated proteolytic activity can have pathological consequences. ADAM10 mediates the shedding of the extracellular domains of a wide range of transmembrane proteins including cell adhesion molecules, cadherins and nectin-1, chemokines, amyloid precursor protein (APP), ephrins and Notch receptors. Hence, ADAM10-mediated shedding of these key proteins plays a major role in the regulation of chemotaxis, inflammation, cell-cell adhesion, and induction of apoptosis [4–6]. Our experiments have shown that ADAM10 also plays an important role in both constitutional and insulin-stimulated shedding of RAGE in macrophages. As a result, ADAM10 can potentially alter RAGE-ligand interaction by cleaving the cell surface receptor and reduce RAGE activation and signaling. Ectodomain shedding of RAGE may therefore be one of the mechanisms involved in the feedback regulation to limit the toxic effects of RAGE-mediated signaling, and targeting ADAM10-mediated shedding of RAGE might have therapeutic potential [3].

In addition, we have further demonstrated that insulin can increase ADAM10 expression and activity. Our findings differed from that of Chen et al. who reported that insulin stimulated ADAM10 proteolytic activity without any demonstrable changes in protein expression [19]. These differences might be partly due to the different experimental conditions used such as the much shorter incubation time in their study. In the present study, we found that the effect of insulin on ADAM10 protein expression was mainly observed at the higher concentrations of insulin. These concentrations are similar to the supraphysiological concentration of insulin seen in the systemic circulation in patients receiving exogenous insulin therapy. Moreover, we have measured ADAM10 activity using a fluorimetric assay and insulin increased both ADAM10 protein expression as well as activity. Insulin may therefore be involved in the regulation of RAGE expression and the production of soluble RAGE. We have previously reported that insulin up-regulates full-length RAGE and esRAGE expression in macrophages [18]. Taken together, this would suggest that insulin not only increases cell surface full-length RAGE expression, it also enhances the ectodomain shedding of RAGE through its effect on ADAM10 expression/activity.

In keeping with our in vitro data, we have found that ADAM10 level was increased in patients with type 1 diabetes compared to non-diabetic controls. To our knowledge, this is the first clinical study investigating ADAM10 level in patients with type 1 diabetes. Whether the increase in ADAM10 level is related to the effect of exogenous insulin therapy remains to be determined. We did not find any association between serum ADAM10 level and insulin dosage of our patients, but total daily insulin dosage is a poor surrogate of plasma insulin level. In addition to having higher serum ADAM10 level, circulating concentrations of sRAGE, esRAGE and cRAGE were also elevated in patients with type 1 diabetes. There was a significant correlation between ADAM10 and cRAGE both in patients with type 1 diabetes and healthy controls, whereas no association between ADAM10 and esRAGE was seen. This is consistent with the way that cRAGE and esRAGE is produced. In the circulation, cRAGE is produced by ectodomain shedding by ADAM10 whereas esRAGE is formed by alternative splicing [11,12]. ADAM10 was an independent determinant of serum cRAGE and our data would suggest that the higher concentration of cRAGE in patients with type 1 diabetes might be partly due to the increase in ADAM10. We speculate that if insulin (especially exogenous insulin) plays a role in regulating the expression and shedding of RAGE, this might partly explains why total sRAGE levels are increased in majority of the studies in type 1 diabetes (reviewed in [20]). Although it has been suggested that soluble forms of RAGE may be used as biomarkers of diabetic vascular complications, it is clear that circulating RAGE isoforms are influenced by many confounding factors including pharmacological agents.

Our study has several limitations. We have only examined the effect of insulin on RAGE shedding by ADAM10 in THP-1 macrophages and we have not examined other cell types like endothelial cells and smooth muscle cells. Our clinical study is a cross sectional study and we cannot prove any causal relationship between ADAM10 and cRAGE, and we have measured only ADAM10 protein level and not activity. In addition, we did not directly assay cRAGE. The assay for sRAGE does not distinguish between the different soluble RAGE species and the assay measures total sRAGEin the circulation. The assay for esRAGE is more specific as the detection antibody in the assay for esRAGE is a rabbit esRAGE-specific polyclonal antibody raised against the unique C-terminal 16-amino-acid peptide (amino acids 332 to 347) of esRAGE. We have used the difference between total sRAGE and esRAGE as a measure of cRAGE because antibodies and corresponding assays for detection of cRAGE are not available. Generation of antibodies specific to cRAGE has so far not been successful. We cannot exclude the possibility there are minor secreted soluble splice variants of RAGE other than esRAGE in the circulation that will be included as cRAGE in our study. However, the contribution is likely to be small. Hudson et al have shown that esRAGE is the main splice variant that is secreted from cells. Most of the other soluble splice variants of RAGE are degraded at mRNA level through nonsense-mediated mRNA-decay pathway and not secreted [12].

In conclusion, we have shown that ADAM10 is involved in constitutional and insulin-stimulated ectodomain shedding of RAGE in vitro. Serum ADAM10 level is increased in type 1 diabetes and there is a significant association between ADAM10 and serum cRAGE level. Our data therefore suggest that ADAM10 might be involved in the regulation of RAGE in diabetes.

## Supporting Information

S1 Fig Effect of insulin on ADAM10 protein expression (A) and shedding of RAGE (B) in human monocyte-derived macrophages (MDMs). Human MDMs were incubated with increasing concentrations of insulin (0 to 50 mIU/ml) or blank medium as control for 24 hours. ADAM10 protein in whole MDMs lysate (A) and shedding of RAGE in MDMs-conditioned media (B) were then measured by Western blot. Data represent the mean ± SEM. *p<0.05 vs control.
(TIF)
