# Supplementary figures and images for: Serum Level of Soluble Receptor for Advanced Glycation End Products Is Associated with A Disintegrin And Metalloproteinase 10 in Type 1 Diabetes
Source: PLoS One. 2015 Sep 1;10(9):e0137330. doi: 10.1371/journal.pone.0137330 (PMC4556489; doi:10.1371/journal.pone.0137330)

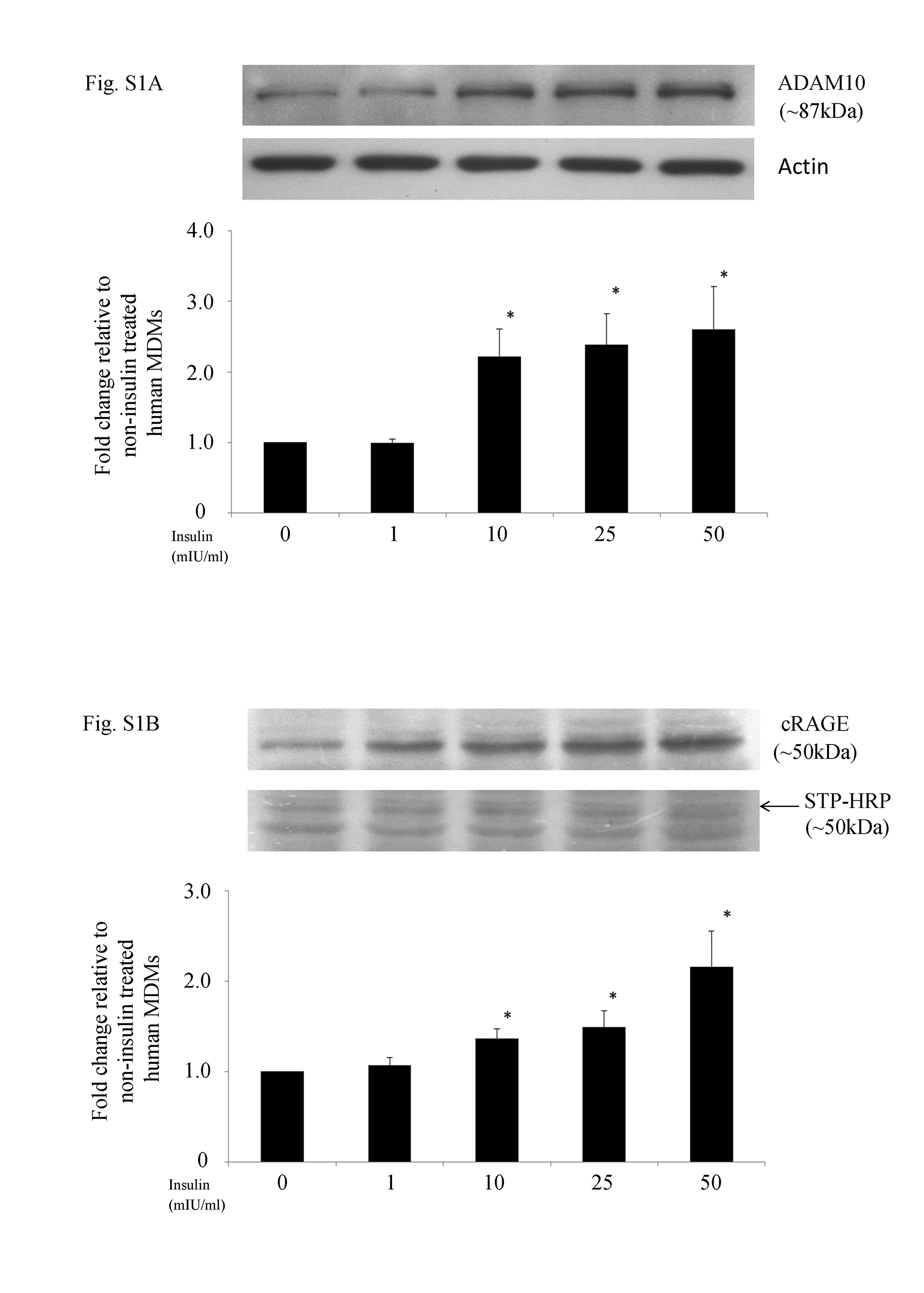

Supplement: S1 Fig — Human MDMs were incubated with increasing concentrations of insulin (0 to 50 mIU/ml) or blank medium as control for 24 hours. ADAM10 protein in whole MDMs lysate (A) and shedding of RAGE in MDMs-conditioned media (B) were then measured by Western blot. Data represent the mean ± SEM. *p<0.05 vs control. (TIF) [file pone.0137330.s001.tif]
